# Loss of PTEN-Induced Kinase 1 Regulates Oncogenic Ras-Driven Tumor Growth By Inhibiting Mitochondrial Fission

**DOI:** 10.3389/fonc.2022.893396

**Published:** 2022-05-05

**Authors:** Dantong Zhu, Fengtong Han, Liuke Sun, Sandeep K. Agnihotri, Ying Hu, Hansruedi Büeler

**Affiliations:** Harbin Institute of Technology, School of Life Science and Technology, Harbin, China

**Keywords:** PTEN-induced kinase-1 (PINK1), mitochondrial metabolism, mitochondrial dynamics, Ras protein, Ras-induced tumors, dynamin-related protein 1 (DRP1), cell cycle

## Abstract

Mitochondrial metabolism and dynamics (fission and fusion) critically regulate cell survival and proliferation, and abnormalities in these pathways are implicated in both neurodegenerative disorders and cancer. Mitochondrial fission is necessary for the growth of mutant Ras-dependent tumors. Here, we investigated whether loss of PTEN-induced kinase 1 (PINK1) - a mitochondrial kinase linked to recessive familial Parkinsonism - affects the growth of oncogenic Ras-induced tumor growth *in vitro* and *in vivo*. We show that Ras_G12D_-transformed embryonic fibroblasts (MEFs) from PINK1-deficient mice display reduced growth in soft agar and in nude mice, as well as increased necrosis and decreased cell cycle progression, compared to Ras_G12D_-transformed MEFs derived from wildtype mice. PINK1 re-expression (overexpression) at least partially rescues these phenotypes. Neither PINK1 deletion nor PINK1 overexpression altered Ras expression levels. Intriguingly, PINK1-deficient Ras-transformed MEFs exhibited elongated mitochondria and altered DRP1 phosphorylation, a key event in regulating mitochondrial fission. Inhibition of DRP1 diminished PINK1-regulated mitochondria morphological changes and tumor growth suggesting that PINK1 deficiency primarily inhibits Ras-driven tumor growth through disturbances in mitochondrial fission and associated cell necrosis and cell cycle defects. Moreover, we substantiate the requirement of PINK1 for optimal growth of Ras-transformed cells by showing that human HCT116 colon carcinoma cells (carrying an endogenous Ras_G13D_ mutation) with CRISPR/Cas9-introduced *PINK1* gene deletions also show reduced mitochondrial fission and decreased growth. Our results support the importance of mitochondrial function and dynamics in regulating the growth of Ras-dependent tumor cells and provide insight into possible mechanisms underlying the lower incidence of cancers in Parkinson’s disease and other neurodegenerative disorders.

## Introduction

Most types of cancers are less common in patients with Parkinson’s disease (PD), but specific tumors may occur more frequently (1, 2) and are associated with mutations or altered expression of familial PD genes (3–5). Although several PD genes act in pathways that protect cells against oxidative stress and mitochondrial dysfunction, they can affect tumor growth in various ways, likely depending on the specific metabolic and cell signaling requirements of different tumor types. For example, DJ-1 acts similar to an oncogene (6, 7), while Parkin has characteristics of a tumor suppressor (8, 9). PINK1 is a mitochondrial kinase linked to recessive familial PD (10). PINK1 phosphorylates ubiquitin on the outer mitochondrial membrane and together with the E3 ligase Parkin promotes the selective degradation of depolarized mitochondria through mitophagy (11, 12).In addition, PINK1 phosphorylates several mitochondrial proteins to increase mitochondrial respiration and regulate mitochondrial dynamics, transport and cellular oxidative stress resistance (13–16). Several studies have linked PINK1 to cancer but its involvement in carcinogenesis is complex and context-dependent, and both pro- and anti-tumorigenic effects of PINK1 have been reported (17–24). Cancer cells undergo complex metabolic rewiring, and increasing evidence shows that many cancers depend on mitochondrial metabolism, signaling and dynamics to promote cancer progression and metastasis, maintain cancer stem cell survival, and confer drug resistance to tumor cells (25, 26). Mutations in the proto-oncogene *RAS* are a frequent cause for a broad spectrum of human cancers (27, 28). Mutant Ras affects mitochondrial function and dynamics in complex ways to promote cell transformation and proliferation (29–31). Because of the central role of PINK1 in mitochondrial function and dynamics, and to further explore the function of PINK1 in cancer, we studied the consequences of PINK1 loss on the growth of oncogenic Ras-driven tumors. Using SV40 large T-immortalized, K-Ras_G12D_-transformed embryonic fibroblasts from PINK1-deficient mice and human HCT116 cells (expressing endogenous Ras_G13D_) with CRISPR/Cas9-induced *PINK1* gene knockout, we show that PINK1 deficiency reduces the growth of tumors expressing oncogenic Ras.

## Results

### PINK1 Deficiency Reduces Growth Rates of RasG12D-Transformed Mouse Embryonic Fibroblasts (MEFs)

**Figure 1:**
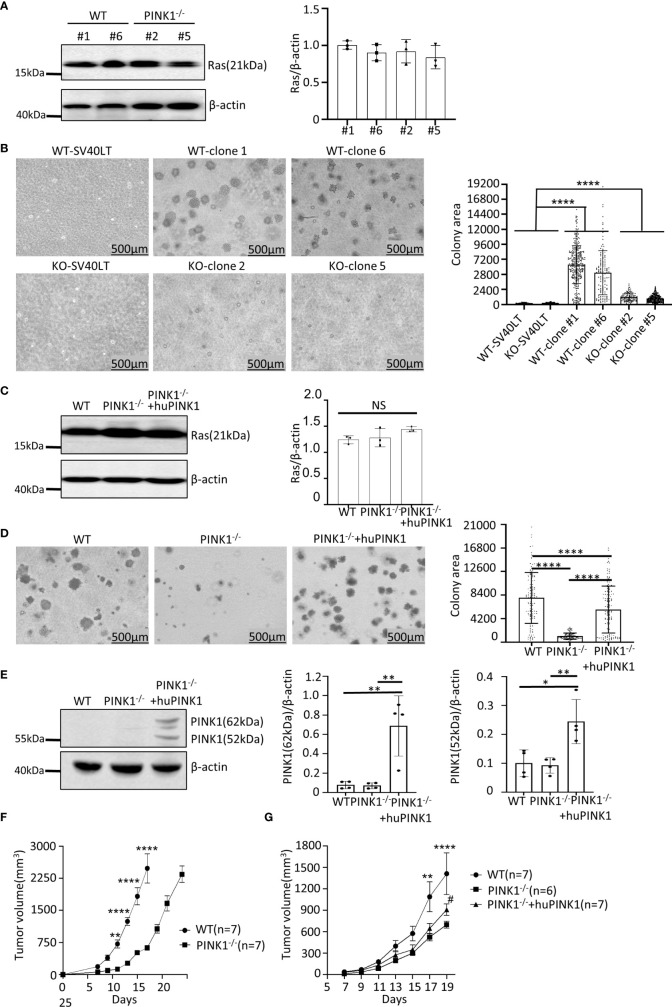
PINK1 deficiency reduces growth of Ras_G12D_-transformed mouse embryonic fibroblasts (MEFs). **(A)** Ras protein expression by western blot with lysates of WT tumor clones 1 and 6 and PINK1^-/-^ tumor clones 2 and 5. **(B)** Image J-measured area of soft agar colonies (mean ± SD, ****p < 0.0001) formed by WT clones 1 and 6, PINK1^-/-^ clones 2 and 5, and immortalized WT and PINK1^-/-^ MEFs that only express SV40LT. Numbers (n) of colonies measured: WT clone 1 = 350; WT clone 6 = 132; PINK1^-/-^ clone 2 = 115; PINK1^-/-^ clone 5 = 292; WT-SV40LT= 106; PINK1^-/–^SV40LT = 305. **(C)** Ras expression in Ras_G12D_-transformed cells populations (n=3 independent protein isolations and western blots). **(D)** Colony area for Ras_G12D_-transformed cells populations (mean ± SD, ****p < 0.0001). Number (n) of analyzed colonies: WT = 128; PINK1^-/-^ = 134; PINK1^-/-^ + huPINK1 = 149). Representative images of colonies are shown. **(E)** PINK1 expression in Ras_G12D_-transformed cells populations (n = 4 independent protein isolations and western blots. 62 kDa (full-length) PINK1 and the 52 kDa PINK1 isoform (product of cleavage by PARL at the inner mitochondrial membrane) are indicated. 62 kDa PINK1: **p = 0.003; 52 kDa PINK1: *p = 0.01; **p = 0.008. **(F)** Tumor volume in nude mice (mean ± SEM, n = 7 mice per genotype/cell population) in experiment terminated on day 17 for WT tumors (due to tumor burden) and on day 24 for PINK1^-/-^ tumors. **(G)** Tumor volume in nude mice (mean ± SEM, n=6-7 mice per genotype/cell population) in experiment terminated on day 19. Stars indicate days with significantly different volumes between WT and PINK1^-/-^ tumors (**p < 0.01, ****p < 0.0001). The # symbol indicates significant different volumes of tumors between PINK1^-/-^ and huPINK1 (p < 0.05). NS, not significant.

To generate Ras_G12D_-transformed cell populations of mouse embryonic fibroblasts (MEFs), we first infected primary MEFs from wild type (WT) and PINK1-deficient mice with a retrovirus expressing simian virus-40 large T antigen (SV40LT), which led to the emergence of continuously growing (immortalized) cells. Subsequently, immortalized MEFs were infected twice with a retrovirus expressing human K-Ras_G12D_ to generate Ras_G12D_-transformed cells, which were used to derive single clones of Ras_G12D_-transformed MEFs by limiting dilution. We selected two single clones with similar SV40LT expression levels for each genotype **(** **Figure S1A** **)**. PINK1 WT clones 1 and 6 and PINK1-deficient clones 2 and 5 expressed comparable levels of Ras protein **(** **Figure 1A** **)**. These four Ras_G12D_-transformed clones were therefore selected for growth comparison in soft agar, which showed that the average colony area for *PINK1^-/-^* clones was about 5-fold smaller than that of the WT control clones (18.8% of WT). No difference in soft agar growth was observed between WT and PINK1-deficient MEFs that only expressed SV40LT **(** **Figure 1B** **)**. We next measured soft agar growth with WT and *PINK1^-/-^* Ras_G12D_-transformed cell populations and also generated a cell population (PINK1^-/-^plus huPINK1) in which human PINK1 was re-expressed in mouse PINK1-deficient Ras_G12D_-transformed cells. Characterization of protein expression showed that Ras protein levels in PINK1^-/-^, WT and PINK1^-/-^plus huPINK1 population cells are similar **(** **Figure 1C** **)**. We also examined the rate of glucose consumption in WT and PINK1^-/-^ Ras_G12D_-transformed cells, and the results showed that MEFs from PINK1^-/-^ Ras_G12D_-transformed had reduced uptake of glucose **(** **Figure S1C** **)**. The average area of PINK1-deficient colonies reached only 13% of that of WT colonies **(** **Figure 1D** **)**. Re-expression of human PINK1 after lentiviral infection substantially rescued the growth deficit, increasing the average colony area to 74% of WT **(** **Figure 1D** **)**. We did not detect PINK1 by western blot in WT Ras_G12D_-transformed MEFs **(** **Figure 1E** **)**, possibly because most of PINK1 is constitutively degraded in normal cells and PINK1 only becomes stabilized upon significant mitochondrial depolarization (11, 32, 33). As expected, PINK1 was absent from PINK1-deficient Ras_G12D_-transformed cells that were derived from MEFs of mice with a null mutation in *PARK6* (34). In contrast, both 62 kDa and 52 kDa PINK1 species were readily detected in PINK1^-/-^plus huPINK1 cells **(** **Figure 1E** **)**, suggesting that human PINK1 was imported to the inner mitochondrial membrane and cleaved by PARL, but that lentiviral over-expression of PINK1 may have saturated its mitochondrial import/cleavage and proteasomal degradation (32, 33).

### PINK1 Deficiency Impairs Tumor Growth of Ras_G12D_-Transformed MEFs in Nude Mice

In nude mice, we injected the same number of MEFs from these two different genotypes (PINK1^-/-^ Ras_G12D_ and PINK1^+/+^ Ras_G12D_). The tumor formation of PINK1^-/-^ cells was significantly reduced when compared with PINK1^+/+^ cells in nude mice. PINK1^-/-^ tumors showed a trend of slower growth at each measurement, and the WT tumors were on average 4.6-fold larger (range: 3.6-5.6) than the PINK1^-/-^ tumors between days 11-17. On day 17, mice with WT tumors were euthanized due to excessive tumor burden, while PINK1^-/-^ tumors were allowed to grow longer until day 24, when they reached similar volumes as the WT tumors had reached on day 17 **(** **Figure 1F** and **Figure S1B** **)**. In agreement with soft agar results, these experiments show that PINK1 deficiency slows the growth of Ras_G12D_-induced tumors in nude mice. Overexpression of PINK1 in the PINK1^-/-^plus huPINK1 population increased tumor growth by day 19 compared to *PINK1^-/-^* Ras_G12D_-transformed cells **(** **Figure 1G** **)**, although the rescue effect was less pronounced than in soft agar (mean tumor volume on day 19: WT = 1413 mm^3^; PINK1^-/-^= 700 mm^3^; PINK1^-/-^plus huPINK1 = 911 mm^3^).

### PINK1 Deficiency Increases Cell Death and Alters Cell Cycle Progression in Ras_G12D_-Transformed MEFs

**Figure 2:**
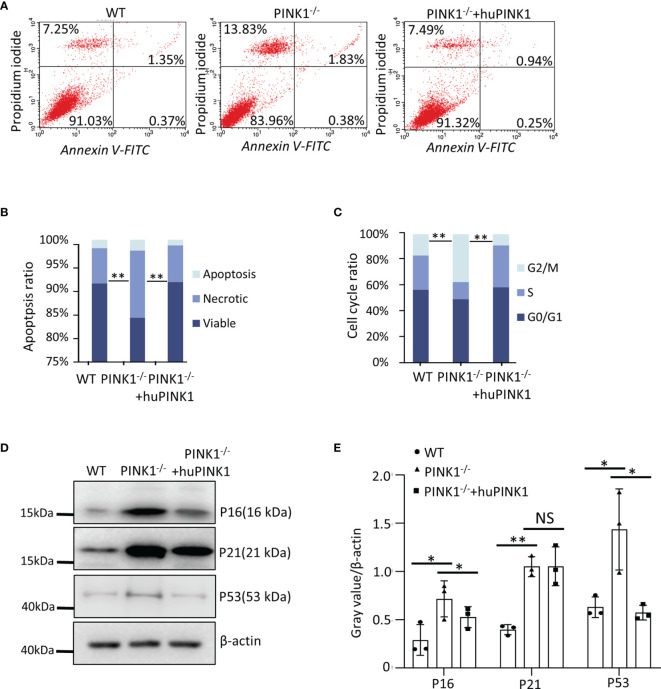
PINK1 deficiency increases cell death and alters cell cycle progression in Ras_G12D_-transformed MEFs. **(A, B)** Apoptotic (Annexin V-FITC+) and necrotic (Propidium iodide+) cells were analyzed by flow cytometry. **(C)** Flow cytometry analysis of the cell cycle. Parts-of-whole columns show percentage of cells in different cell cycle phases. For each genotype/population, cells were plated into n = 3 wells, which were separately stained with propidium iodide and analyzed by flow cytometry (**p < 0.01, ANOVA). **(D, E)** Western blot for P16, P21 and P53 of WT, PINK1^-/-^ and PINK1^-/-^ +huPINK1 cells (n = 3 independent protein isolations, *p < 0.05, **p < 0.01). NS, not significant.

We further asked whether the reduced growth of PINK1-deficient tumors is due to increased cell death or decreased cell cycle progression, as both are hallmarks of cancer and involved in Ras-induced tumorigenesis. Cell death was measured with Annexin V/propidium iodide (PI) staining assays in RasG12D-transformed MEFs with different genetic backgrounds. As shown, PINK1 loss increased the percentage of necrotic but not apoptotic cells, which was fully reversed by re-expression of human PINK1 **(** **Figures 2A, B** **)**. Consistent with the function of PINK1 in maintaining mitochondrial integrity and its roles in regulating cell death, PINK1^-/-^ cells displayed reduced membrane potential (Δψm) **(** **Figure S2A** **)**. There was a positive correlation between Δψm and mean colony area in soft agar **(** **Figure S2B** **)**. In addition, total ROS and mitochondrial ROS were reduced in PINK1^-/-^ cells. Recovery PINK1 expression at least partially restored the reduced ROS in PINK1^-/-^ cells **(** **Figures S2D, E** **)**. Furthermore, basal mitochondrial respiration was reduced in PINK1^-/-^ RasG12D-transformed MEFs as revealed by Seahorse analysis **(** **Figure S2C** **)**. These data suggest that mitochondrial metabolism is changed by PINK1.

Cell cycle analysis of PI-stained cells by FACS demonstrated that PINK1 deficiency led to an increase of Ras_G12D_-transformed cells in the G2/M phase and decrease of cells in the G0/G1 phase. Overexpression of PINK1 had the opposite effect, reducing the percentage of cells in G2/M and increasing the proportion of cells in G0/G1 **(** **Figure 2C** **)**. In line with these results, protein levels of p16 and p21 were increased in PINK1^-/-^ cells by Western blot analysis **(** **Figures 2D, E** **)**. In addition, p53 protein expression was increased in PINK1^-/-^ Ras_G12D_-transformed cells **(** **Figures 2D, E** **)**. These data suggest that loss of PINK1 promotes cell death and cell cycle arrest.

### The Impact of PINK1 on Cell Death and Cell Cycle Progression Is Validated *In Vivo* in Nude Mice

**Figure 3:**
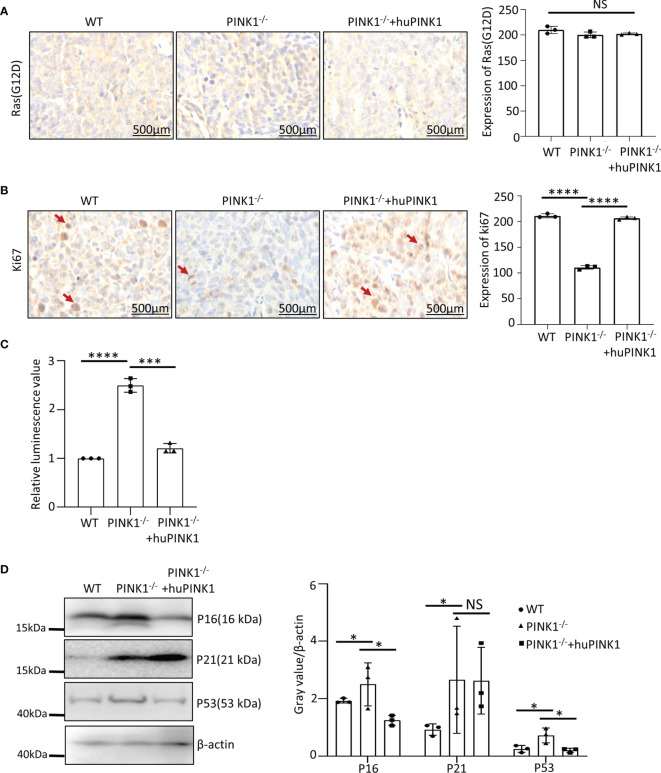
Impact of PINK1 deletion on cell death and cell cycle progression are validated *in vivo* in nude mice. **(A)** Immunohistochemical analysis of WT, PINK1^-/-^ and PINK1^-/-^ +huPINK1 tumor tissue was performed using RasG12D antibody. Image gray value is analyzed by Image J (n = 3 immunohistochemical images of tumor tissue). **(B)** Immunohistochemical analysis of WT, PINK1^-/-^ and PINK1^-/-^ +huPINK1 tumor tissue with Ki67 antibody. Image gray value is analyzed by Image J (n = 3 immunohistochemical images of tumor tissue, ****p < 0.0001). **(C)** Detection of apoptosis in WT, PINK1^-/-^ and PINK1^-/-^ +huPINK1 tumor tissue detected by the caspase 3/7 kit. (n = 3 tumor tissues, ***p < 0.001, ****p < 0.0001). **(D)** Western blot for P16, P21 and P53 of WT, PINK1^-/-^ and PINK1^-/-^ +huPINK1 tumor tissue (n = 3 tumor tissue protein lysates, *p < 0.05). NS, not significant.

To study the mechanisms underlying PINK1-regualted tumor growth *in vivo*, we examined cell proliferation rates in tumor tissues derived from WT, PINK1^-/-^ and PINK1^-/-^plus huPINK1 cells in nude mice. Ras_G12D_ protein levels were comparable in the various tumor populations **(** **Figure 3A** **)**. In contrast, expression of the cell proliferation marker Ki67 in PINK1^-/-^ tumors was reduced compared to WT, while expression of Ki67 in PINK1^-/-^plus huPINK1 tumors that over-expressed huPINK1 was increased, indicating reversible inhibition of tumor cell proliferation *in vivo* by ablation of PINK1 **(** **Figure 3B** **)**. Thus, the reduced growth of PINK1-deficient Ras_G12D_-transformed cells *in vitro* is validated and supported by reduced proliferation of the corresponding tumor cells in nude mice.

Using the caspase 3/7 apoptosis detection kit, we also observed increased apoptosis of PINK1^-/-^ tumor cells in nude mice **(** **Figure 3C** **)**. Expression levels of p16, p21 and p53 were significantly increased in PINK1^-/-^ tumor tissues, which was reversed after re-expression of human PINK1. However, unlike in MEFs, p21 was not downregulated significantly after re-expression of human PINK1 **(** **Figure 3D** **)**.

### PINK1 Deficiency Impairs Mitochondrial Fission in Ras_G12D_-Transformed MEFs

**Figure 4:**
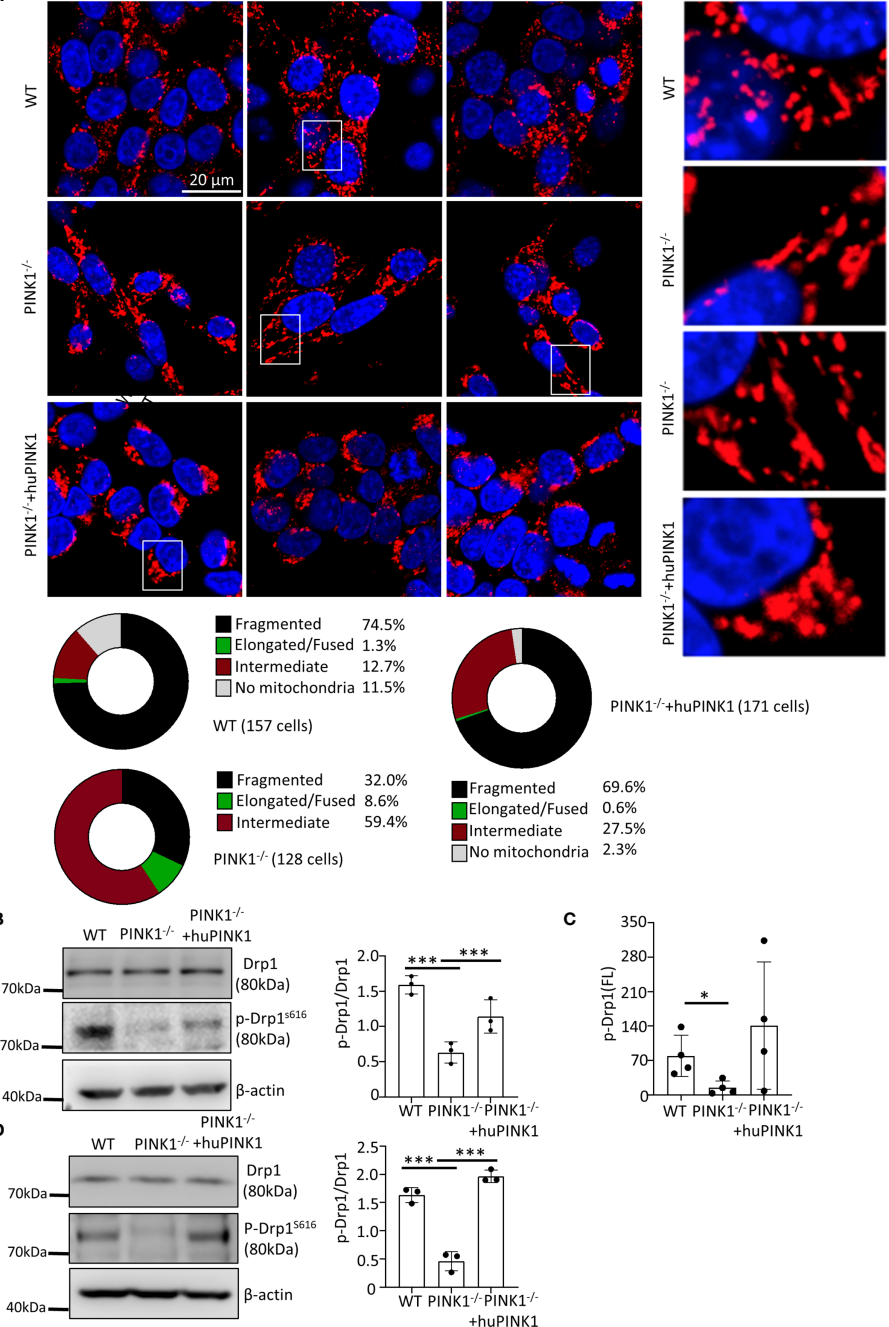
PINK1 deficiency impacts mitochondrial fission in Ras_G12D_-transformed MEFs. **(A)** Cells were stained with an antibody against LRP130, which is exclusively expressed in the matrix of mitochondria. Nuclei were visualized with DAPI. Three representative images for each genotype of cells are shown, with magnifications of the mitochondria within white rectangles displayed on the right side. Bottom: Mitochondrial network morphology was analyzed as described in the Methods, and the percentage of cells with fragmented, intermediate, and elongated/fused mitochondrial networks, or no mitochondria, is indicated for WT, PINK1^-/-^ and PINK1^-/-^ +huPINK1 cell populations. Number of cells analyzed: WT = 157, PINK1^-/-^ = 128; PINK1^-/-^ +huPINK1 = 171. **(B)** Expression and phosphorylation of Drp1 in WT, PINK1^-/-^ and PINK1^-/-^ +huPINK1 cells by western blots (n = 3 cell protein lysates). **(C)** Expression and phosphorylation of Drp1 in WT, PINK1^-/-^ and PINK1^-/-^ +huPINK1 cells analyzed by flow cytometry (n = 3, mean ± SD, *p < 0.05). **(D)** Expression and phosphorylation of Drp1 in WT, PINK1^-/-^ and PINK1^-/-^ +huPINK1 tumor tissue analyzed by western blots (n = 3 cell protein lysates). ***p < 0.001.

We next explored possible mitochondrial mechanisms underlying the reduced growth of PINK1-deficient Ras_G12D_-transformed cells. As indicated above, PINK1 deletion did not influence Ras expression levels (**Figure 1C**). Several studies have shown that PINK1 acts as a pro-fission factor (14, 35–38), consistent with mitochondrial fission preceding mitophagy (36, 39) and the enlarged mitochondrial morphology in various cells of PINK1-deficient mice (40). To analyze whether loss of PINK1 altered mitochondrial morphology, we stained mitochondria in MEFs by immunocytochemistry with an antibody against LRP130. LRP130 is a suitable marker because it is exclusively expressed in the mitochondrial matrix where it regulates the assembly and activity of cytochrome c oxidase (complex IV) (41). The mitochondrial network was highly fragmented in the majority (74.5%) of WT Ras_G12D_-transformed cells **(** **Figure 4A** **)**. In contrast, only 32% of *PINK1^-/-^* Ras_G12D_-transformed cells showed a fragmented mitochondrial network, while 59.4% showed an intermediate network and 8.6% showed an elongated/fused network **(** **Figure 4A** **)**. Re-expression of PINK1 restored mitochondrial fragmentation to 69.6% of the cells, similar to that in WT cells **(** **Figure 4A** **)**. Thus, loss of PINK1 interfered with mitochondrial dynamics in Ras_G12D_-transformed MEFs, which could be reversed by re-expression of PINK1.

Because it has been shown that PINK1 promotes mitochondrial fission by phosphorylating Drp1 at Ser616 (42), we studied whether altered mitochondrial dynamics in PINK1^-/-^ Ras_G12D_- MEFs was related to abnormal phosphorylation or expression of Drp1. To this end, the expression of Drp1 and phosphorylated Drp1 was detected by western blotting and flow cytometry. There was no significant difference in Drp1 protein expression. PINK1^-/-^ MEFs showed a significant decrease of phospho-Drp1 (Ser616) levels compared to WT MEFs. Re-expression of huPINK1 in PINK1^-/-^ cells partially rescued the decreased phospho-Drp1 (Ser616) levels **(** **Figures 4B, C** **)**. Analysis of the expression of Drp1 and phosphorylated Drp1(Ser616) showed that phosphorylation of DRP1 was also decreased in PINK1^-/-^ tumor tissues **(** **Figure 4D** **)**. It has been also shown that ERK can increase Drp1 phosphorylation (43). However, our data did not show a reliable link between ERK phosphorylation (activation) and PINK1 expression **(** **Figure S2G** **)**, suggesting that decreased phosphorylation at Drp1 (Ser616) may be due to a direct effect of PINK1 loss. In contrast to the phosphorylation at Drp1(ser616), phosphorylation at Drp1(Ser637) inhibits mitochondria fission and modulate mitophagy (44). Interestingly, PINK1 deficiency did not cause alterations in Drp1(Ser637) phosphorylation **(** **Figure S2F** **)**. In line with above data, although PINK1 deficiency increased p62 and LC3-1/II, the recovery of PINK1 expression had no ability to abolish it **(** **Figure S2H** **)**, suggesting that the increased p62 and LC3-1/II is not intrinsic effect of PINK1. Therefore, PINK1 deficiency regulate mitochondria morphology mainly by reducing Drp1(Ser616) phosphorylation-mediated mitochondria fission.

Drp1 affects mitochondrial fission, leading to cell cycle arrest and apoptosis (45–47), which prompted us to explore the impact of PINK1 on cell growth in the presence of the mitochondrial fission (Drp1) inhibitor Mdivi-1. Mdivi-1 significantly inhibited the growth of Ras_G12D_-transformed WT cells, while the inhibitory effect of Mdivi-1 was compromised in PINK1-deficient cells. Re-expression of PINK1 in PINK1^-/-^ Ras_G12D_ MEFs restored Mdivi-1-mediated growth inhibition (**Figure S3A**). Therefore, PINK1 loss inhibits tumor growth at least in part through impaired regulation of Drp1. Together with data presented above, these results demonstrate a pro-fission and pro-growth function of the PINK1-Drp1 axis in Ras_G12D_-transformed MEFs.

### PINK1 Knockout Reduces the Growth of HCT 116 Human Colon Carcinoma Cells *In Vitro*

In MEFs, Ras_G12D_ was overexpressed after viral transduction. To study whether PINK1 loss similarly affected the growth of authentic human Ras_G13D_-transformed tumor cells in which mutated Ras is expressed at physiological levels, we introduced CRISPR-Cas9-mediated deletions into the *PINK1* gene of HCT116 cells that were originally isolated from a primary human colon carcinoma. Several PINK1-deficient HCT116 clones with varying *PINK1* deletions were identified by PCR with genomic DNA using primers and DNA sequencing that flanked the two CRISPR target sites **(** **Figures S3B, C** **)**. We did not detect PINK1 protein expression by Western blots in WT HCT116 cells, likely due to constitutive degradation in cells without severe mitochondrial depolarization (32). However, in addition to sequencing, we confirmed that the CRISPR/Cas9-generated genomic *PINK1* deletions produced null mutations at the mRNA level by PCR amplification of total cellular cDNA with two different primers pairs **(** **Figure S4A** **)**. PINK1-knockout clones 45 and 51 produced the expected PCR fragments (deletions), which were unable to encode any functional PINK1 protein. In addition, clones 3, 11 and 50 yielded no bands or only very faint PCR products, suggesting that in these clones the genomic deletions destabilized the resulting PINK1 mRNAs to levels below detection. Collectively, these results show that all CRISPR/Cas9-generated *PINK1* deletions produced null mutations.

**Figure 5:**
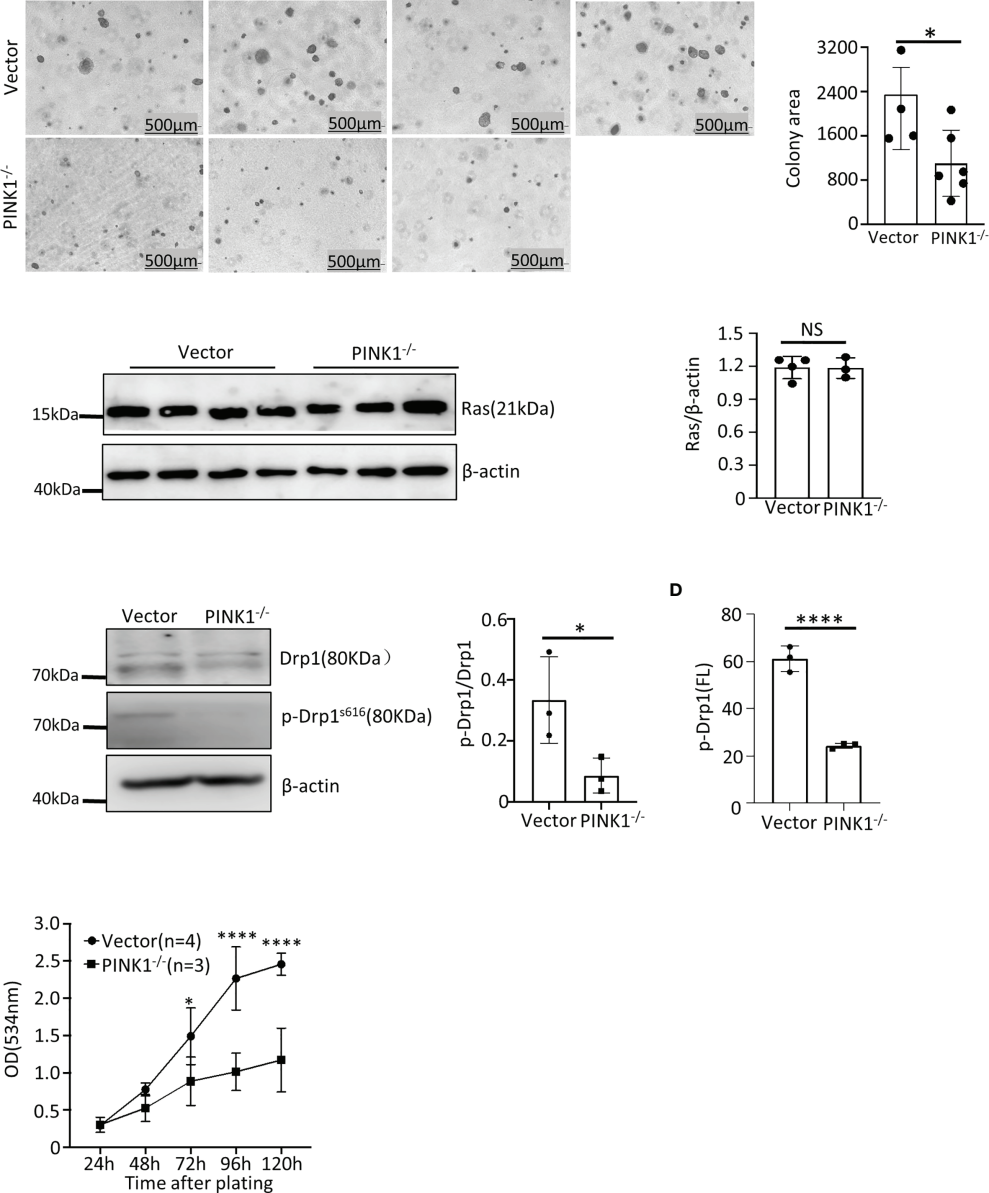
PINK1 knockout reduces the growth of HCT 116 human colon carcinoma cells *in vitro*. **(A)** Left: Representative images of soft agar colonies with vector and PINK1^-/-^ genotype. Right: Graph comparing average colony area (mean ± SD) of 4 control vector-transfected HCT116 clones and 6 CRISPR-generated PINK1-knockout HCT116 clones (*p = 0.047). **(B)** Western blot quantification of Ras expression in the vector and PINK1^-/-^ HCT116 cell clones (n = 3 clones per genotype). **(C)** Expression and phosphorylation of Drp1 vector-transfected and PINK1^-/-^ cells by western blots. **(D)** Phosphorylation of Drp1 in vector and PINK1^-/-^ HCT116 clones by flow cytometry (n=3 clones, mean ± SD, ****p < 0.0001). **(E)** Cell number (growth) of HCT116 control and PINK1^-/-^ cell clones measured in tissue culture with the sulforhodamine B assay (mean ± SD, two-way ANOVA, *p = 0.037 and ****p < 0.0001).

HCT116 clones lacking PINK1 showed reduced growth in soft agar when compared to the control clones, which were transfected with px458 vector only **(** **Figure 5A** **)**. This was not due to altered Ras expression, because PINK1-deficient and control vector-transfected HCT116 clones expressed comparable levels of Ras **(** **Figure 5B** **)**. Deletion of PINK1 also did not affect the GTPase activity of Ras (**Figure S4B**). Like in MEFs, we detected Drp1 and phosphorylated Drp1(Ser616) protein in HCT116 cells by western blotting and flow cytometry and found a decrease in phosphorylated Drp1(Ser616) in PINK1 knockout HCT116 cells **(** **Figures 5C, D** **)**. In tissue culture, growth of PINK1 knockout HCT116 cells was significantly slower than that of the control group **(** **Figure 5E** **)**. Overall, these results show that in human tumor cells with a common Ras mutation, the loss of PINK1 also causes impaired tumor growth.

### PINK1 Ablation Affects Mitochondrial Fission and Increases Cell Death in HCT-116 Cells

**Figure 6:**
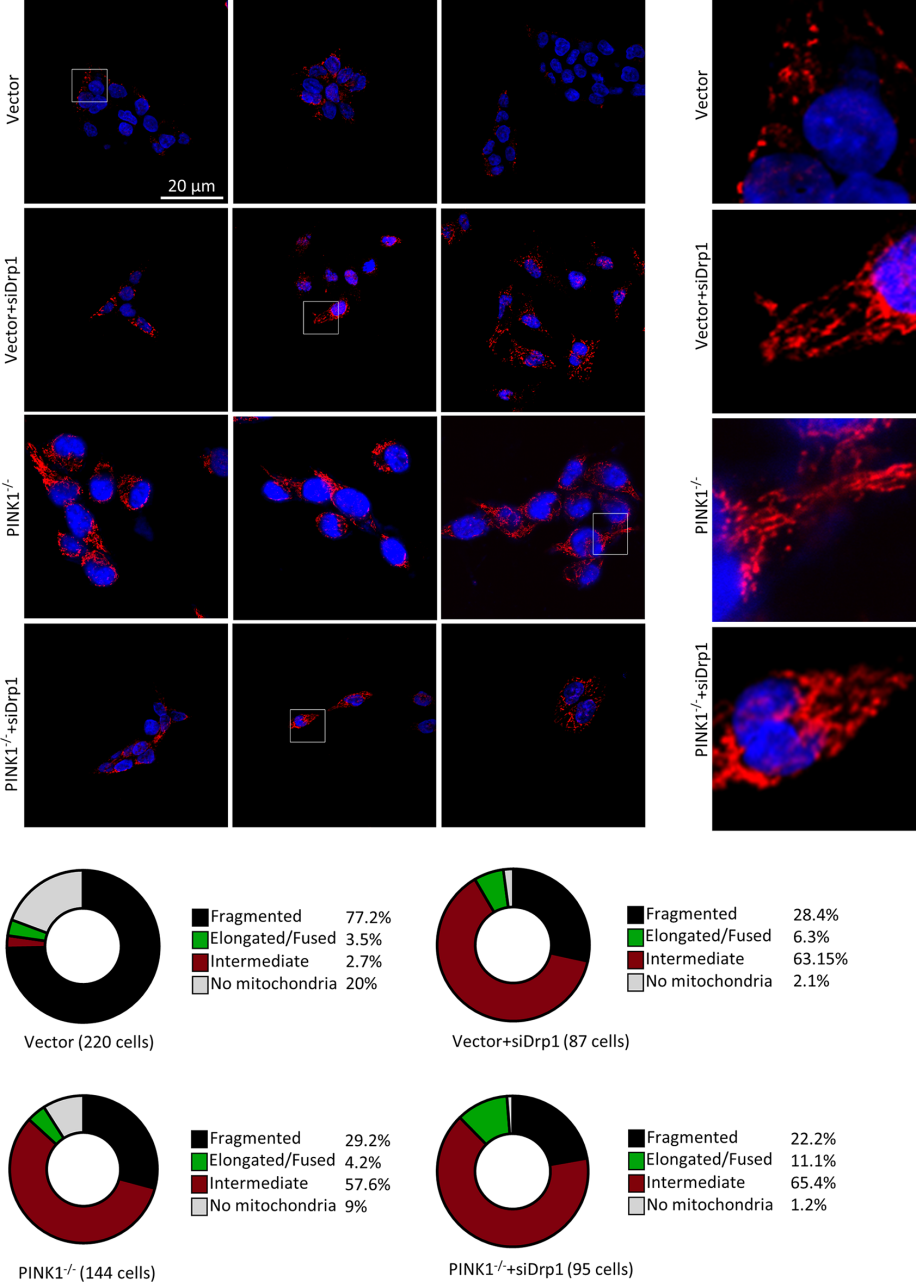
PINK1 knockout affects mitochondrial fission in HCT116 cells. Cells were stained with an antibody against LRP130, which is exclusively expressed in the matrix of mitochondria, and nuclei were visualized with DAPI. Three representative images for each genotype of cells, with magnifications of the mitochondria within white rectangles displayed on the right side. Bottom: Mitochondrial network morphology was analyzed as described in the Methods, and the percentage of cells with fragmented, intermediate, and elongated/fused mitochondrial networks, or no mitochondria, is indicated for vector, vector+siDrp1, PINK1-/-and PINK1^-/-^+siDrp1. Number of cells analyzed: Vector = 220, Vector+siDrp1 = 87, PINK1^-/-^ = 144, PINK1^-/-^+siDrp1 = 95. NS, not significant.

Similar to the results reported in other studies that most of the mitochondria were fragmented in cell lines containing Ras mutations (48), mitochondrial fragmentation was also observed in HCT116 vector-transfected cells. In contrast, HCT116 PINK1 knockout cells showed significantly less fragmentation with a shift to intermediate-size mitochondria. Specifically, PINK1^-/-^ cells contained 29.2% fragmented mitochondria compared with 77.7% in the vector group **(** **Figure 6** **).** It has been reported previously that upon Drp1 deletion, the mitochondrial morphology in HCT116 cells was more intermediate (48–50). In our study, we inhibited Drp1 expression in HCT116 cells by small interfering RNA (siRNA), and the knockdown efficacy was determined by western blot **(** **Figure S4C** **)**. Followed by confocal microscopy analysis of mitochondrial morphology. We observed a decrease in intracellular mitochondrial fragmentation after Drp1 knockdown (KD) in the control cells. However, in PINK1^-/-^ HCT116 cells, mitochondrial morphology did not change much and more of it was still in fusion state **(** **Figure 6** **)**. This suggests that the effect of PINK1^-/-^ on mitochondrial morphology is similar to that of Drp1 reduction.

**Figure 7:**
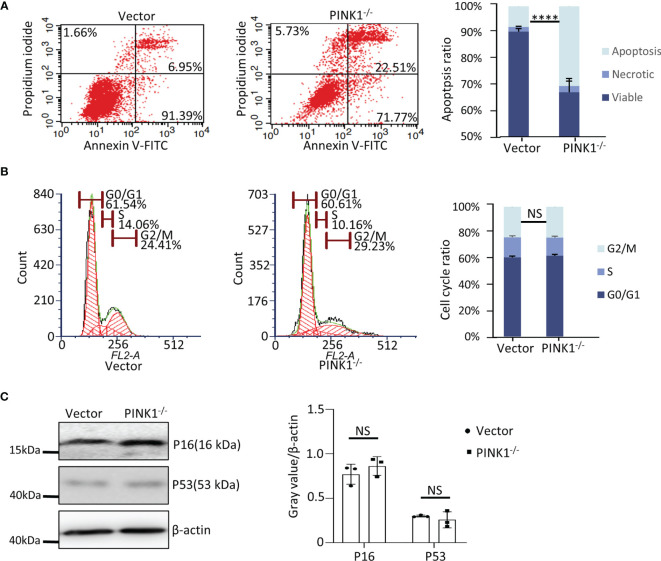
PINK1 knockout affects cell death in HCT116 cells. **(A)** Apoptotic (Annexin V-FITC+) and necrotic (Propidium iodide+) cells of vector and PINK1^-/-^ HCT116 clones were analyzed by flow cytometry (n = 3 clones per genotype, mean ± SD, ****p < 0.0001). **(B)** Flow cytometry analysis of the cell cycle. Representative cell cycle profiles of vector and PINK1^-/-^ are depicted. Parts-of-whole columns show percentage of cells in different cell cycle phases. For each genotype, cells were separately stained with propidium iodide and analyzed by flow cytometry (n = 3 clones per genotype). **(C)** Western blot for P16 and P53 expression in vector and PINK1^-/-^ cells (n=3 clones per genotype). NS, not significant.

It has been shown previously that deletion of Drp1 increases apoptosis (49). Analyzing apoptosis in HCT116 cells, we found that 22.51% of the PINK1^-/-^ cells underwent apoptosis while only 6.95% of the WT cells were apoptotic **(** **Figure 7A** **)**. Thus, similar to MEFs, cell death was also increased in PINK1^-/-^ HCT116 cells, although in MEFs increased death was mainly due to increased necrosis rather than apoptosis. In HCT116 cells, we also used PI to study the cell cycle. In contrast to MEFs, we found no difference in the percentage of cells in the G2/M phase in HCT116 cells lacking PINK1**(** **Figure 7B** **)**. In agreement with this result, expression of the cell cycle-related checkpoint proteins p16 and p53 was unaltered in PINK1^-/-^ HCT116 cells **(** **Figure 7C** **)**.

## Discussion

Oncogenic *RAS* mutations are a frequent cause of many human tumors (estimated 30%), especially cancers of the pancreas, lung and colon (27, 28). Here we show that loss of PINK1 reduces tumor cell growth in two different Ras-driven tumor models: Embryonic fibroblasts (MEFs) from *PINK1^-/-^* mice immortalized with SV40 large T and transduced with a Ras_G12D_ retrovirus, and patient-derived HCT116 human colon carcinoma cells expressing an endogenous Ras_G13D_ mutation with CRISPR-Cas9-introduced *PINK1* gene deletions. We cannot entirely exclude that a subtle difference in Ras expression may have contributed to reduced tumor cell growth in the MEF system. However, the fact that two individual *PINK1^-/-^* Ras-transformed MEF clones that expressed Ras protein at levels comparable to two WT clones were severely growth-impaired, strongly supports an important role for PINK1 in the growth of Ras-driven tumors. This conclusion is corroborated by the observation that PINK1 ablation also reduced the growth of several Ras_G13D_-transformed human HCT116 tumor cell clones – without decreasing Ras protein expression or Ras GTPase activity. Finally, PINK1 loss failed to reduce the growth of immortalized MEFs that only expressed SV40LT but not Ras_G12D_. Taken together, these results demonstrate that PINK1 ablation does not generally reduce cell growth but that PINK1 expression is required for optimal growth of Ras-transformed cells.

Mitochondrial dynamics supports different stages of tumorigenesis (51). Mitochondrial fission (or fragmentation) is required for Ras-induced carcinogenesis (48, 52, 53). Intriguingly, we found that PINK1 deletion impairs mitochondrial fragmentation in Ras_G12D_-induced MEF cells and HCT116 cells containing an endogenous Ras_G13D_ mutation. This suggests that PINK1 may support mutant Ras-mediated tumorigenesis by stimulating fission to facilitate tumor growth. Indeed, PINK1 and Parkin promote fission and/or inhibit fusion (14, 35–38), and we have previously shown that elongated and enlarged mitochondria accumulate in several primary cell types from PINK1-deficient mice (40). Drp1 affects mitochondrial morphology and is critical for mitochondrial fission in mammalian cells (54, 55). We also show that PINK1 deficiency impairs Drp1 phosphorylation in tumor cells, as has been reported previously in other cells (42). Reduced levels of phospho-Drp1 (Ser616) in *PINK1^-/-^* tumor cells shown here, and impaired PINK1/Parkin-mediated proteasomal degradation of mitofusin (36, 56–58), may have contributed to increased mitochondrial fusion in *PINK1^-/-^* tumor cells. We also show that PINK1 deletion compromised the ability of the Drp1 inhibitor Mdivi-1 to reduce the growth of Ras-transduced cells, supporting that PINK1 acts at least in part by regulating Drp1 to inhibit tumor growth, likely *via* PINK1-mediated phosphorylation of Drp1. However PINK1 may also support Ras-induced tumorigenesis by Drp1-independent mechanisms.

**Figure 8:**
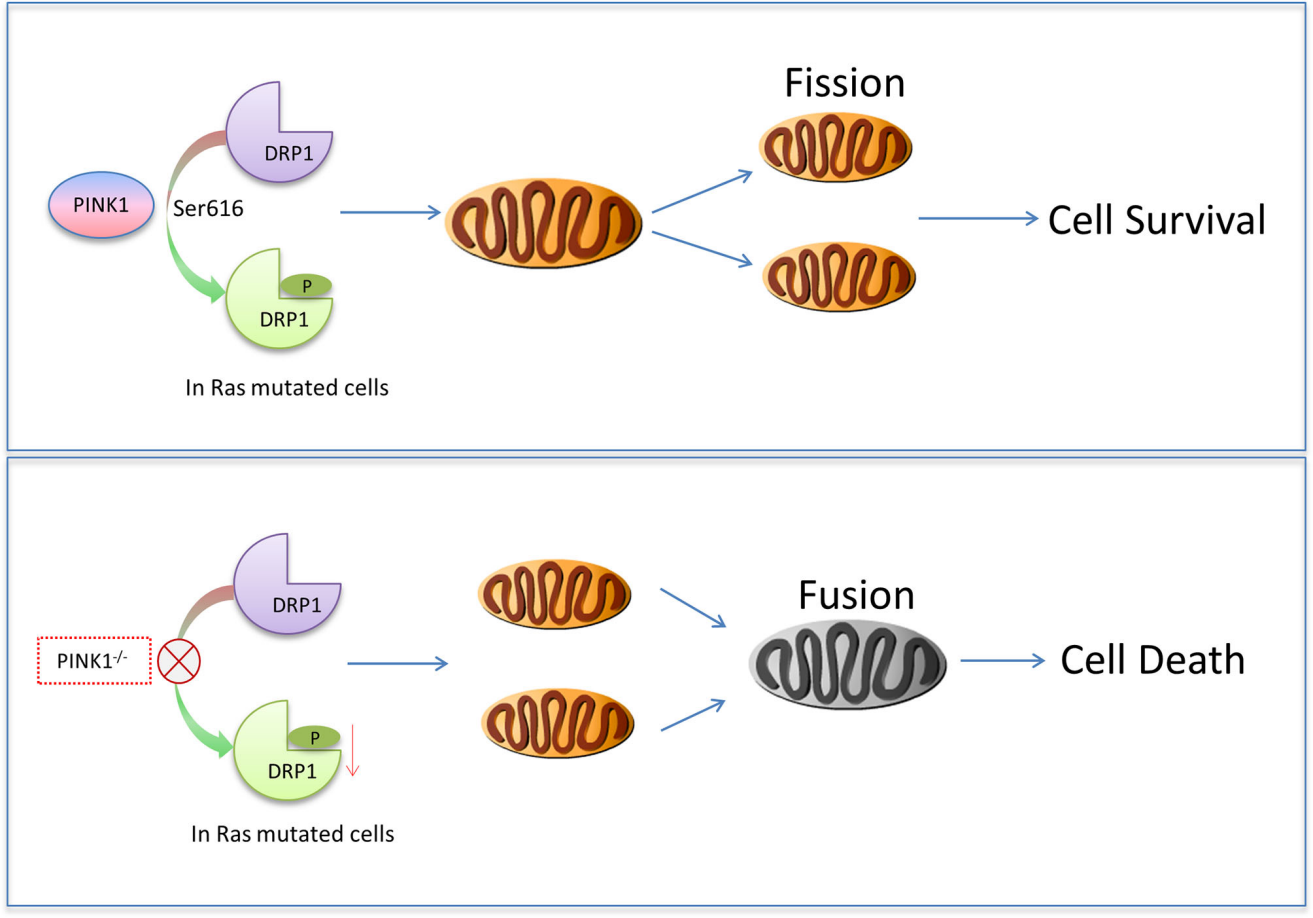
Model of how PINK1 may cause cell death in mutant Ras-transformed tumor cells. In cells with Ras mutations that contain endogenous PINK1, Drp1 S616 is phosphorylated for mitochondrial fission to promote cell survival. When PINK1 was absent, Drp1 S616 was not phosphorylated, more mitochondrial fusion occurred, and cells with Ras mutation died more.

Mitochondrial dynamics regulates cell cycle progression, and blocking mitochondrial fission interferes with the completion of mitosis (43, 54, 59–61). It was therefore interesting to study cell cycle progression in PINK1-deficient Ras_G12D_-transformed cells with aberrant mitochondrial dynamics. The shift toward increased mitochondrial fusion in *PINK1^-/-^* MEF Ras_G12D_-transformed cells may explain the accumulation of these cells in G2/M, as it is observed in tumor cells after Drp1 knockdown (61). In addition, PINK1 phosphorylates Drp1 at Ser616 which stimulates fission (42), and we show here impaired phosphorylation of Ser616 of Drp1 in PINK1-deficient Ras_G12D_-transformed cells. In contrast, PINK1 overexpression decreased the percentage of cells in G2/M and increased the proportion of cells in G0/G1. Because mitochondrial hyper-fusion is necessary for progression from G1 into S phase (60), PINK1-stimulated mitochondrial fission (14, 37) in cells overexpressing PINK1 likely accounts for the accumulation of cells in G0/G1 in the PINK1^-/-^plus huPINK1 population. Similar cell cycle defects have been described for SV40LT-immortalized mouse embryonic fibroblasts lacking PINK1 (20), showing that mitotic defects due to PINK1 loss are not limited to Ras_G12D_-transformed cells. However, the mechanisms of mitotic arrest may be different in Ras_G12D_ expressing cells, because cellular signaling and context not only influences mitochondrial dynamics but also determines the consequences of abnormal mitochondrial dynamics on cell proliferation and death (59). Consistent with the cell cycle defects, p16, p21, and p53 were elevated in PINK1-deficient MEF cells. PINK1 overexpression rescued impaired mitochondrial fission and reversed cell cycle deficits, consistent with the importance of mitochondrial dynamics for cell cycle regulation (31, 43, 54, 59, 60). Taken together, these results suggest that aberrant mitochondrial dynamics due to impaired phosphorylation of Drp1 and associated cell cycle and cell survival defects may be primary mechanisms underlying reduced tumor cell growth in PINK1^-/-^ MEF cells (**Figure 8**).

It should be noted that in the human colon cancer cell line HCT116, deletion of *PINK1* mainly increased apoptosis of tumor cells but had no effect on G2/M cell cycle arrest. In contrast, PINK1-deficient Ras-transformed MEFs showed increased necrosis, possibly through the aging pathway (62). Because HCT116 is a mature human tumor cell line, various pathways are likely different from those in Ras-transformed engineered MEFs.

## Conclusions

Our results corroborate the importance of mitochondrial function and dynamics in promoting the growth of Ras-dependent tumors and provide insights into possible mechanisms underlying the lower incidence of cancers in Parkinson’s disease and other neurodegenerative disorders. Because a single inhibitor or antineoplastic agent against PINK1 has great limitations, future studies are warranted and necessary to determine whether reducing PINK1 expression, especially in combination with chemical anti-tumor agents (63–66), could constitute a viable approach to combat mutant Ras-induced and other types of cancers. In addition, although our work does not place PINK1 in a Ras-dependent transformation pathway, separate projects addressing such a possibility with additional transformed tumor models may be interesting.

## Experimental Procedures

### Animals

*PINK1^-/-^* mice have been described previously (34) and were backcrossed for at least 15 generations onto the pure C57BL/6J background. Male nude mice (BALB/c-nu, 4-6 weeks old) were purchased from Beijing Vital River Laboratory Animal Technology, China. Animal experiments were performed according to the “Guide for the Care and Use of Laboratory Animals”, 8^th^ Edition, 2011 (The National Academic Press, Washington, and D.C.) and approved by the Institutional Animal Care and Use Committee/Animal Experimentation Ethics Committee of the Harbin Institute of Technology (HIT-IACUC).

### Oligonucleotides, Antibodies and Tissue Culture Media

Oligonucleotides **(** **Table S1** **)** and antibodies **(** **Table S2** **)** are listed in Supporting Information. We used antibodies at the manufacturer-recommended concentrations, and obtained tissue culture media, trypsin and phosphate-buffered saline (PBS) from Thermo Fisher and fetal bovine serum (FBS) from JYK Biotechnology, China.

### Construction and Production of Recombinant Viral Vectors

Retroviral vectors expressing SV40LT, EGFP and human K-Ras_G12D_ were based on the vector SFG-MCS, a derivative of the plasmid MFG (67). To generate recombinant retroviruses, we transfected 10 μg viral vector DNA per 6-cm plate into PlatE packaging cells (68) using the calcium phosphate co-precipitation method. To produce lentiviral particles for PINK1 re-expression, we inserted the human PINK1 coding sequence into the lentiviral vector pLVSIN-CMV-Pur (Takara Biotech) and co-transfected 293T cells in 6-cm plates with 5 μg lentiviral vector plasmid, 3.33 μg HIV gag/pol expression plasmid psPAX2 (Addgene #12260) and 1.67 μg VSV-G expression plasmid pMD2.G (Addgene # 12259) (3:2:1 ratio). Viral particles in the medium were collected 24 and 48 hours after transfection, passed through a 0.45 μm filter and stored at -80°C.

### Generation of Ras_G12D_-Transformed MEF Tumor Clones and Populations

We isolated mouse embryonic fibroblasts (MEFs) from E14.5 stage embryos of WT and *PINK1^-/-^* mice as described (69). For immortalization, we infected MEFs with SFG-SV40LT retrovirus in the presence of 4 μg/ml polybrene (Sigma H9268). The immortalized phenotype was confirmed by unlimited passage ability, in contrast to MEFs infected with SFG-EGFP retrovirus that stopped dividing several passages after infection. For transformation, we infected SV40LT-immortalized MEFs twice separated by 24 hours with SFG-Ras_G12D_ retrovirus in presence of 4 μg/ml polybrene to yield a Ras_G12D_-transformed population. Individual Ras_G12D_ transformed tumor clones were obtained by limiting dilution in 96-well plates. To express human PINK1 in mouse PINK1-deficient Ras_G12D_-transformed cells, we infected cells with the pLVSIN-PINK1-Pur lentiviral vector and selected stably transduced cells by growth in medium containing 2 μg/ml puromycin (Sigma P8833).

### CRISPR/Cas9-Mediated Generation and Characterization of PINK1-Deficient HCT116 Tumor Cells

HCT116 cells were obtained from American Type Culture Collection (ATTC, CCL-247). We used a modified version of the CRISPR-Cas9 plasmid pSpCas9(BB)-2A-GFP (PX458) (Addgene 48138) that was engineered to contain a puromycin expression cassette and BbsI and BsaI cloning sites for insertion of two double-stranded oligonucleotides encoding different sgRNAs. The oligonucleotides encoding sgRNAs targeting two sites in *PINK1* exon 1 are shown in **Table S1**. After transfecting the CRISPR/Cas9 PINK1 targeting vector into HCT116 cells, puromycin-resistant (2 μg/ml) single clones were obtained by limiting dilution. Genomic DNA from clones was PCR-amplified with primers PINK1-KO-screen-fw and PINK1-KO-screen-rev to identify clones that produced a single, shorter PCR product compared to wildtype genomic DNA **(** **Figure S3B** **)**. PCR products from such clones were sequenced and the sequences aligned with the human *PINK1* gene (gene ID: 65018) to determine the exact *PINK1* deletions in different HCT116 PINK1-knockout clones **(** **Figure S3C** **)**. The two CRISPR target sites, clone-specific deletions and PCR primers used to identify PINK1-knockout clones are indicated in **Table S1**. In all clones, the deletions resulted in severely truncated proteins containing only 72 to 113 N-terminal amino acids before running into a premature stop codon, thereby lacking the entire PINK1 kinase domain. To analyze deletions in PINK1 mRNA, cDNA was synthesized from total RNA (Prime Script cDNA kit, Takara Bio) and PCR-amplified with two different primer pairs (fw-1/rev-1 and fw-1/rev2) flanking the deletions **(** **Figure S4A** **)**.

### Growth of Tumor Cells in Soft Agar and Tissue Culture

Cells were grown in DMEM with 10% FBS. Before the soft agar assay, 1.5x10^4^ cells were resuspended in 0.35% low-melting point agarose (Sigma) in DMEM/10% FBS and plated into triplicate wells of a 6-well plate containing a 2 ml base agarose layer (0.7%). After 8-10 days, images of cells were taken with a Nikon inverted microscope at 200x magnification. Three random images per well were captured at different soft agar depths (9 images total), and the colony areas were measured with Image J software (imagej.nih.gov/ij). To measure the growth of HCT116 tumor clones in tissue culture, we plated 10^4^ cells per clone and well (in triplicate wells) and measured cell numbers with the sulforhodamine B assay (70) over a period of 5 days. The average OD534 from three wells was statistically compared between PINK-1 deficient and control tumor clones using 2-way ANOVA.

### Growth of Tumor Cells in Nude Mice

Cells were detached with trypsin, resuspended in DMEM/10% FBS and centrifuged for 5 min at 500 x *g*. The cell pellet was washed twice in PBS and cells were counted in a hemocytometer (four big squares). Cell density was adjusted to 1.25x10^7^ or 2.5x10^7^ cells/ml, and 200 μl of cell suspension (2.5x10^6^ or 5x10^6^ cells, matched for all genotypes) was injected subcutaneously into mice. Tumor growth was monitored starting at 7 days and dimensions of tumors were measured with a caliper at the days indicated in the figures. Tumor volumes (in mm^3^) were calculated as length x width^2^ x 0.5 (width being the smaller dimension) and compared using two-way ANOVA. At the end of the experiment mice were euthanized by CO_2_ inhalation.

### Quantification of SV40LT mRNA With Real-Time qPCR

Cellular RNA was isolated with Trizol reagent, and first strand cDNA synthesized with the Prime Script RT kit (Takara Inc.) from 500 ng total RNA. Two μl of the resulting cDNA (5-fold dilution) was subjected to real-time PCR using SYBR Premix Ex Taq II (Tli RNase H Plus) master mix (Takara Inc). PCR primers are listed in **Table S1**. Melting curve analysis was done to confirm single PCR products. We used the 2^-ΔCt^ method (71) to calculate mRNA expression of each gene relative to 18S rRNA.

### Quantification of Proteins by Western Blots

Cells were lysed in modified RIPA buffer (50 mM Tris-HCl pH 8.0, 1 % Triton X-100, 0.1 % SDS, 0.14 M NaCl, 1 mM EDTA, and 1 mM EGTA) containing 1% (v/v) protease inhibitor cocktail and - where appropriate - phosphatase inhibitor cocktail (Amresco). 20-30 μg total proteins in the cleared lysates (supernatants of 10 min, 12,000 x *g* centrifugation) were analyzed by standard Western blot procedures. Secondary antibodies were conjugated to fluorophores or HRP, and protein bands were detected with the Odyssey Infrared Imaging System (Li-COR) or the enhanced chemiluminescence imager. Protein bands were quantified using Image J software (imagej.nih.gov/ij). Three to four independent cell lysates (prepared at different passage numbers) of each genotype (MEF population) and the indicated number of WT and PINK1-deficient cell clones were analyzed and used for statistical evaluation.

### Flow Cytometry

To measure expression of total Drp1 and phospho-Drp1 (Ser616) by flow cytometry, 5x10^5^ cells were fixed in 4% PFA for 30 min and centrifuged at 500 x *g* for 5 min. The cell pellet was gently resuspended and incubated in 100 μl PBS/0.2% Triton X-100 for 10 min, washed with PBS, and the cells were incubated with primary antibody diluted in PBS/0.05% Tween-20 (PBST) for 1 hour at RT (antibody dilutions as recommended by the manufacturer). After washing twice in PBST, the cells were incubated for 30 min with 1:400-diluted Cy3-conjugated secondary antibody, followed by a final wash and re-suspension in PBST before flow cytometry. To determine background fluorescence and set the fluorescence window for positive cells (M1), control cells were incubated with secondary antibody only. N=4 independent antibody incubations/flow cytometry experiments were carried out with each genotype/cell population for statistical analysis. To measure the mitochondrial membrane potential Δψm, cells were incubated with 50 nM TMRE for 30 min at 37°C. Δψm was compared between groups of WT and PINK1^-/-^ cells, where the groups were comprised of individual cell clones and the cell population for each genotype (n=3-4 clones/populations per genotype, each clone and population measured in triplicate). Mitochondrial ROS were measured in WT, PINK1^-/-^ and PINK1^-/-^plus huPINK1 (human PINK1-overexpressing) cell populations incubated for 30 min at 37°C with 2.5 μM MitoSOX Red (ThermoFisher M36008) (n=3 dye incubations/flow cytometry experiments per genotype/population). Negative control cells were incubated in buffer without TMRE or MitoSOX to determine background fluorescence and set the window for positive cells (M1). Fluorescence (FL2-H) was measured with the BD FACSCalibur using a 585/42 nm band pass emission filter, and the mean fluorescence of the cells in the positive window (M1) was compared between genotypes/cell populations.

### Analysis of Mitochondrial Morphology

For analysis of mitochondrial network morphology, cells (4x10^5^/35-mm plate) grown on cover glasses were washed with PBS, fixed in 4% PFA and mitochondria were stained by immunocytochemistry with anti-LRP130 antibody and Cy3-conjugated secondary IgG. LRP130 is exclusively expressed in mitochondria (41). Nuclei were visualized with DAPI. The percentage of cells with various mitochondrial morphologies was analyzed in a blinded manner using 15 random confocal images per genotype (87-243 cells; 5-13 images from 3 cover glasses), taken with 40x objective and digitally enlarged 3x for analysis using Image J software. Cells with mostly (>80-90%) small and spherical (fragmented) mitochondria were classified as having a “fragmented” mitochondrial network, cells with appreciable proportions (>40-50%) of elongated/fused mitochondria that also contained fragmented mitochondria were classified as “intermediate”, and cells with mostly (>80-90%) elongated/fused mitochondria were classified as “elongated/fused".

### Cell Cycle Analysis

Cells (10^5^/cm^2^) were grown for 48 hours before being collected by trypsinization and resuspended in ice-cold 70% ethanol for fixation (1 hour on ice). Fixed cells were centrifuged (400 x *g* for 5 min), washed once with 2 ml PBS and centrifuged again. The final cell pellet was resuspended in PBS containing 1:10 diluted propidium iodide/RNase A solution (Sungene Biotech, China), and cells were incubated for 30 min at RT and protected from light. Cells were analyzed by flow cytometry (FACSCalibur, Becton Dickinson) and data files were imported into FCS Express 6.0 Plus software (*De Novo* software, Glendale, CA). For processing and quantification, the main cell population was gated in the FSC vs. SSC plot. Within the main population, cell debris and aggregated cells were excluded by gating on single cells in the FL2-A vs. FL2-W plot. Markers for G0/G1, S and G2/M phases were placed in the FL2-A vs. cell count plot of WT Ras_G12D_-transformed cells, and the same markers were used for all other cell samples.

### Immunohistochemical Analysis

Tumor tissues dissected from nude mice fixed with 10% formalin, and embedded in paraffin. The tissue sample was subjected to antigen retrieval by boiling in 0.01 mol/L citrate buffer for 5 min. Slides were then incubated with anti-RasG12D or anti-Ki-67 antibody at 4°C. for the night. Slides were then washed with PBS, fixed with 10% formalin and provided for staining. Detection was carried out by the REAL EnVision detection system (Dako) with diaminobenzidine peroxidase serving as chromogen. Images were collected, and were de-interfered and then quantitatively analyzed for gray values using Image J software.

### Caspase 3/7 Activity Apoptosis Assay

The apoptotic rates were evaluated by Caspase-Glo^®^ 3/7 Assay kit (Promega, G8090). Briefly, the tumor tissues were harvested in urea buffer (2M Thiourea, 4% CHAPS, 40mM Tris-Base, 40mM DTT, 2% Pharmalyte) and sonicated to crush DNA. The same amount of tumor tissues protein lysates (500μg) was transfer into the 96-well cell culture plate. Caspase 3/7 activity assay was repeated independently for three times according to its manufacturer’s instructions, which was represented as a fold-increase of fluorescence calculated by comparing to vector groups.

### Ras Activation Assay

The small GTPase activity of Ras was measured using the Ras activation assay Biochem kit (cytoskeleton #BK008). Cells were harvested with cell lysis buffer (50 mM Tris pH 7.5, 10 mM MgCl2, 0.5 M NaCl, and 2% Igepal when reconstituted) contains 1×Protease Inhibitor Cocktail on ice. The resulting protein lystates were immediately clarified by centrifugation at 10,000 rpm, for 2 min at 4°C and protein concentrations were determined by Bradford kit. After that, 400ug of protein lysates was incubated with Raf-RBD beads (Cat. # RF02) on a rotator at 4°C for 1h. The Raf-RBD beads were pelleted by centrifugation at 3-5,000 × g for 1 min at 4°C. 90% of the supernatant was carefully removed and the beads were washed once with 500μl Wash Buffer. The Raf-RBD beads were pelleted again by centrifugation at 3,000xg for 3 min at 4°C. The supernatant was carefully removed without disturbing the beads. 20 μl of 2×Laemmli sample buffer was added into each tube and the beads were boiled for 2 min. Then resulting lysates were subjected to the following western blot analysis of Ras.

### Quantification of Cell Death (Apoptosis and Necrosis)

Apoptotic and necrotic cells were detected by the Annexin V-FITC apoptosis analysis kit (Tianjin Sungene Biotech, China). Different genotypes of cells density were adjusted to 5 × 10^6^ cells/ml. About 100 μl cell suspension was incubated with 2.5 μl AnnexinV/FITC for 10 min and then 2.5 μl PI for 5 min at room temperature in dark. The rate of apoptosis was measured by flow cytometry (BD Pharmingen).

### Statistics

Statistics was performed with Prism 9.0 software (Graph Pad). All data are presented as mean ± SD, except tumor volumes in nude mice, which are shown as mean ± SEM. Two groups were compared by unpaired *t*-test, and three or more groups were compared by ANOVA and Tukey’s multiple comparisons test. Two-way ANOVA with Sidak’s multiple comparisons test was used to compare tumor growth in nude mice and the growth of WT and PINK1-knockout HCT116 clones in culture. Differences were considered significant at *P*<0.05.

## Data Availability Statement

The original contributions presented in the study are included in the article/**Supplementary Material**. Further inquiries can be directed to the corresponding authors.

## Ethics Statement

The animal study was reviewed and approved by Harbin Institute of Technology.

## Author Contributions

DZ contributed to the main experimental data. MEF immortalized cell lines were prepared by FH and LS provided immunofluorescence images. SA assisted tumor injection and dissection in nude mice. YH and HB are my supervisors, who supervised the overall development of this manuscript and provided funds and laboratory support. All authors contributed to the article and approved the submitted version.

## Funding

This work was supported by a grant from the China National Science Foundation (31970715) to HB.

## Conflict of Interest

The authors declare that the research was conducted in the absence of any commercial or financial relationships that could be construed as a potential conflict of interest.

## Publisher’s Note

All claims expressed in this article are solely those of the authors and do not necessarily represent those of their affiliated organizations, or those of the publisher, the editors and the reviewers. Any product that may be evaluated in this article, or claim that may be made by its manufacturer, is not guaranteed or endorsed by the publisher.
