# Supplementary material for: Loss of PTEN-Induced Kinase 1 Regulates Oncogenic Ras-Driven Tumor Growth By Inhibiting Mitochondrial Fission
Source: Front Oncol. 2022 May 5;12:893396. doi: 10.3389/fonc.2022.893396 (PMC9117651; doi:10.3389/fonc.2022.893396)
Supplement: Supplementary file 1 [file DataSheet_1.docx]

**Supporting Information**

**Loss of PTEN-induced kinase 1 regulates oncogenic Ras-driven tumor growth by inhibiting mitochondrial fission**

**Dantong Zhu^1^, Fengtong Han^1^, Liuke Sun^1,2^, Sandeep K. Agnihotri^1,3^, Ying Hu^1,^* and Hansruedi Büeler^1,^***


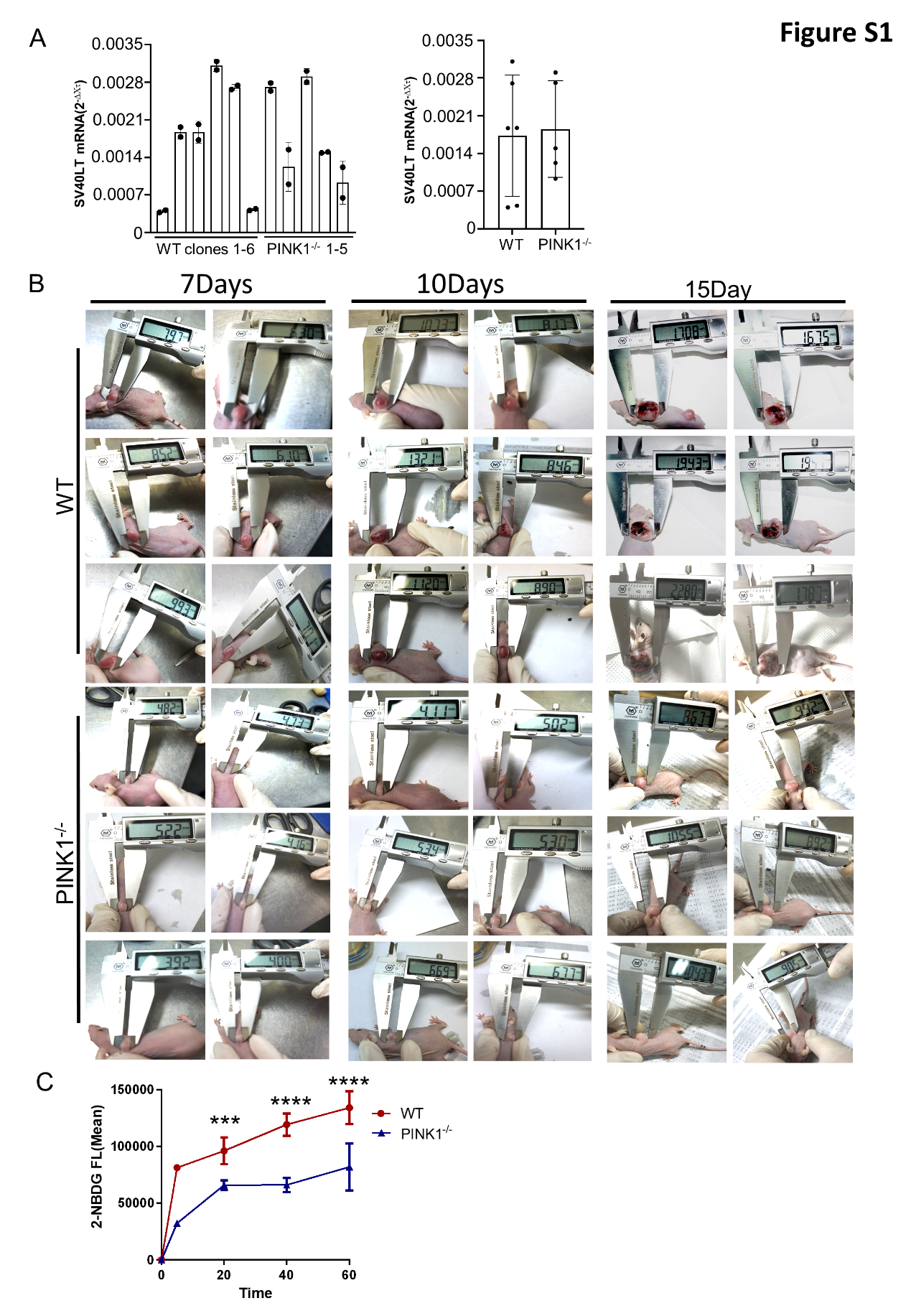


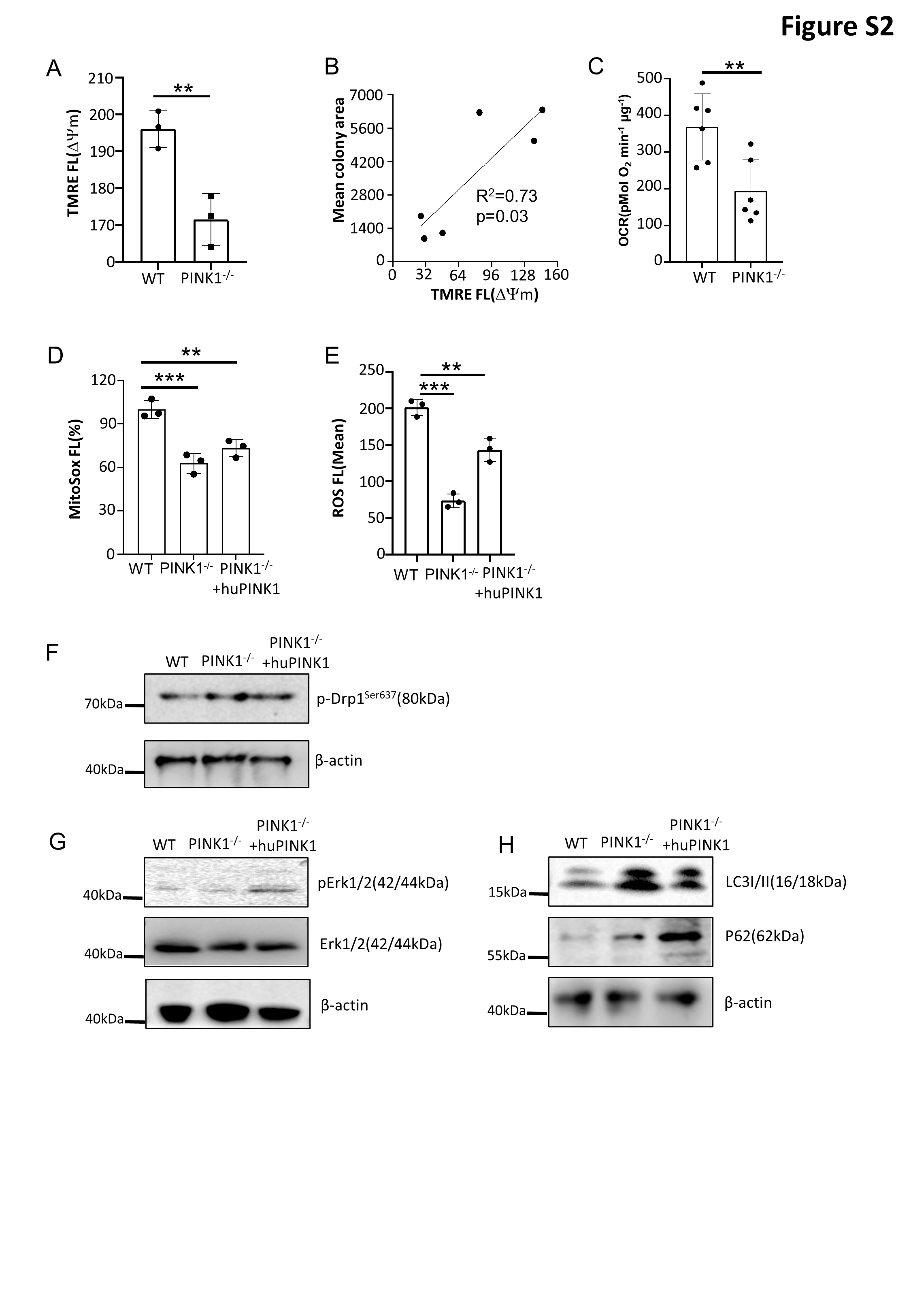


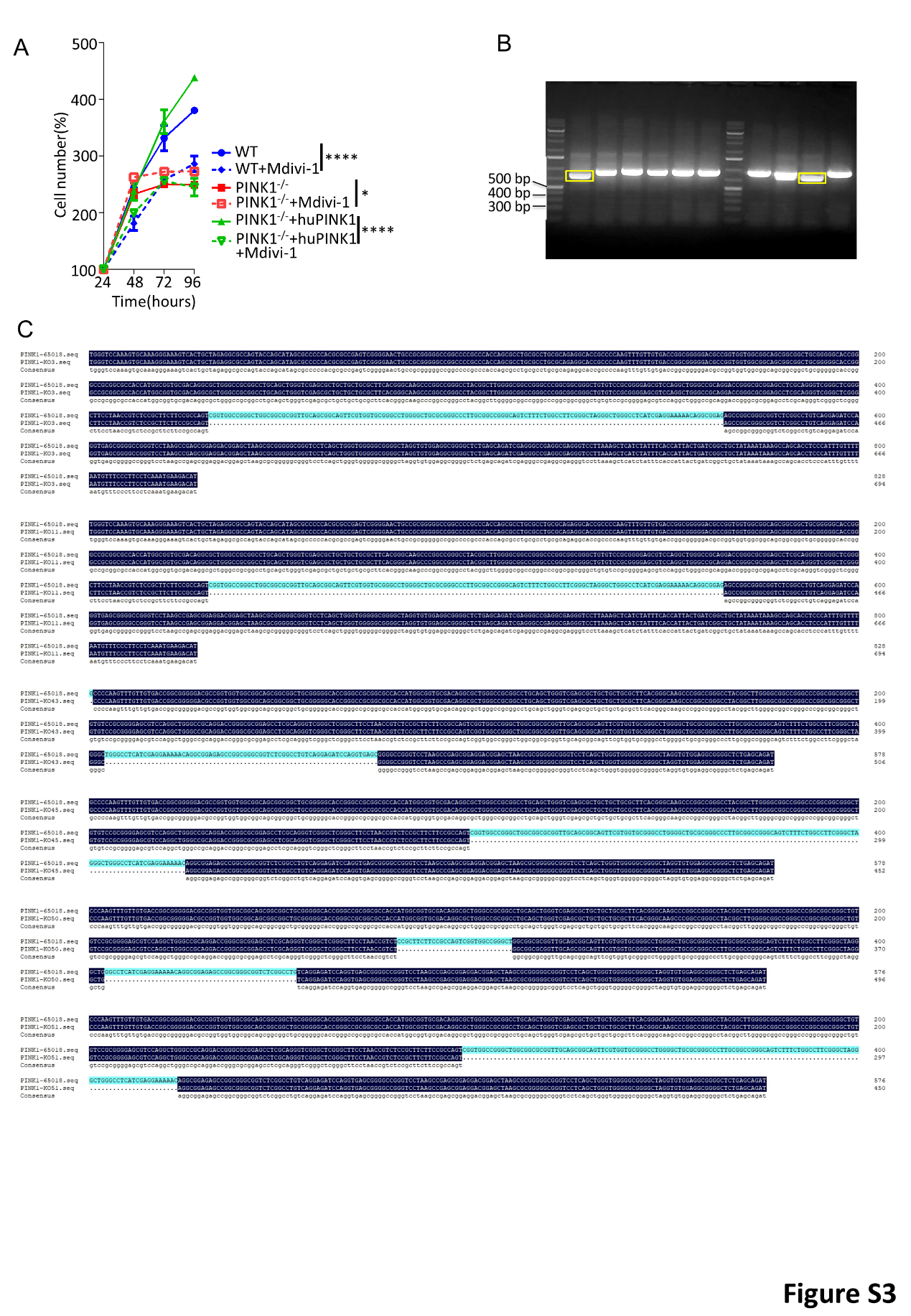


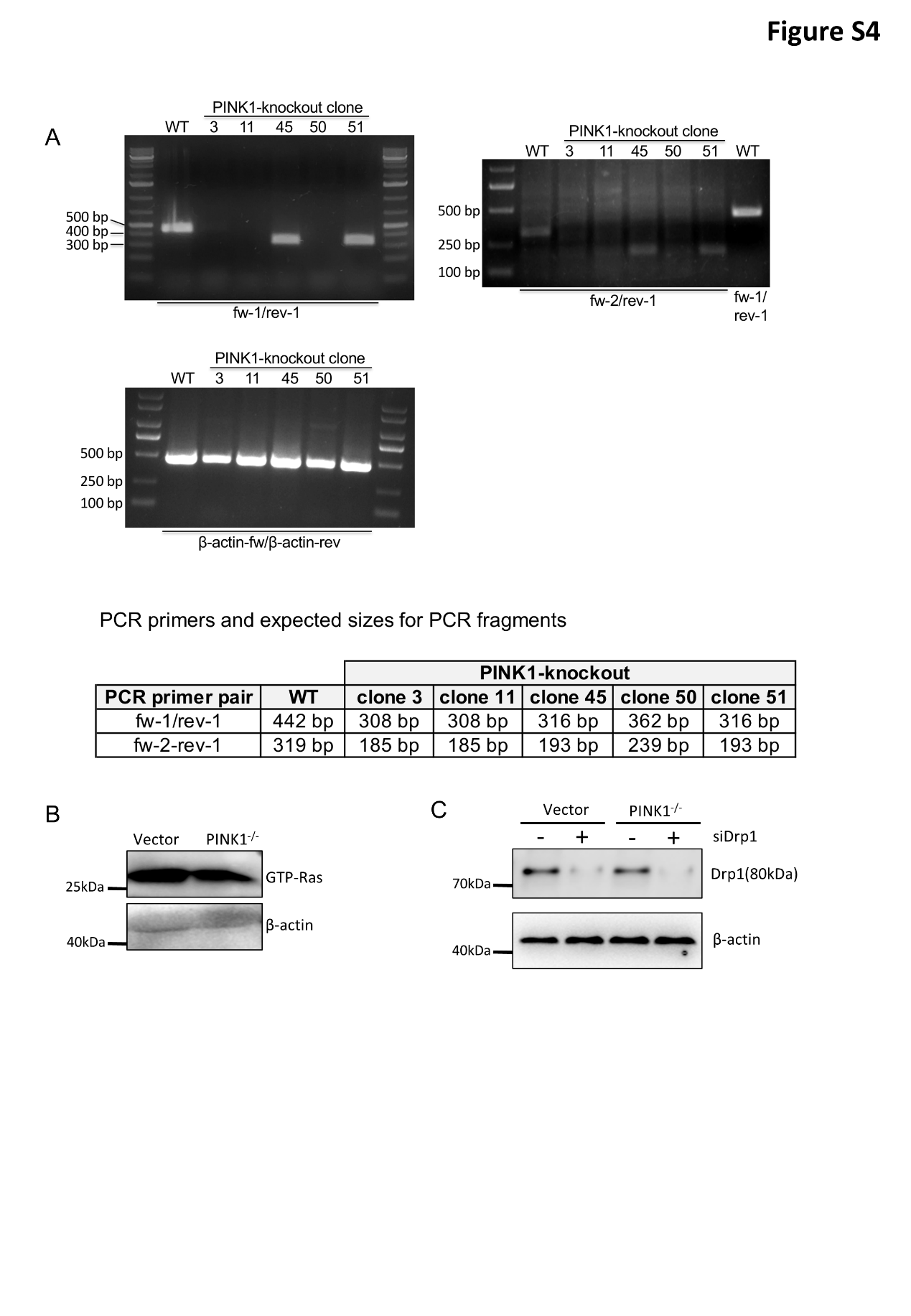


**Figure S1.** (A) Expression of SV40LT mRNA in WT clones 1-6 and PINK1-/- clones 1-5. The graphs on the left show mRNA expression in individual clones (mean ± SD of duplicate qPCR reactions), while graphs on the right show the mean ± SD for all clones of each genotype (n=6 for WT; n=5 for PINK1^-/-^). (B) Images of WT and PINK1^-/-^ cells injected at 7, 10 and 15 days in nude mice. (C) 2NBDG glucose uptake was measured by flow cytometry. （n=3 independent samples）. ***p=0.0004, ****p＜0.0001.

**Figure S2.** (A)Δψm was measured as TMRE fluorescence in flow cytometry(n=3 experiments per genotype/cell population, **p=0.0079). (B) Six of the seven tumor cell lines shown in panel A were also analyzed in soft agar growth assays, and Pearson correlation shows that the Δψm of individual tumor cell lines correlates with their growth in soft agar (R2 =0.73, p=0.03). (C) Basal mitochondrial respiration (oxygen consumption rate, OCR) measured by extracellular flux analysis (XF24e Seahorse analyzer) in WT and PINK1^-/-^ tumor cell populations (n=6 wells per genotype from two experiments, **p=0.006). (D)Mitochondrial ROS was measured as MitoSOX fluorescence in flow cytometry (n=3 experiments per genotype/cell population, ***p=0.0009, **p=0.005). (E) ROS was measured fluorescence in flow cytometry. (n=3 experiments per genotype/cell, ***p＜0.0005, **p＜0.005). (F) Expression of phosphorylation of Drp1 Ser637 in WT, PINK1^-/-^ and PINK1^-/-^ +huPINK1 MEFs analyzed by western blots (n=3 cell protein lysates). (G) Expression of ERK and p-ERK in WT, PINK1^-/-^ and PINK1^-/-^ +huPINK1 MEFs analyzed by western blots (n=3 cell protein lysates). (H) Expression of p62 and LC3I/II in WT, PINK1^-/-^ and PINK1^-/-^ +huPINK1 MEFs analyzed by western blots (n=3 cell protein lysates).

**Figure S3.** (A) Growth of tumor populations in absence and presence of 30 μM Mdivi-1 (mitochondrial fission inhibitor) shows that Mdivi-1 effectively inhibits the growth of KrasG12D-driven cancer cells expressing PINK1, while it fails to do so in PINK1-deficient cells. N=3 per tumor population (condition) and time point (mean ± SEM, ****p＜0.0001，*p=0.0246). Growth of cells is expressed relative to the 24h time point (baseline, 100%) for each genotype/treatment combination to avoid potential confounding effects of PINK1 loss on cellular NADH levels (measured by the CCK-8 kit). (B-C) The image is PCR identification of PINK1 knockout and Alignments of individual PINK1-knockout clone sequences (genomic PCR product) with the human PINK1 gene (<https://www.ncbi.nlm.nih.gov/gene/65018>).

**Figure S4.** (A) Image of PCR-amplified with two different primer pairs (fw-1/rev-1 and fw-1/rev2) flanking the deletions. (B) Ras GTP activity detected by western blot. Vector and PINK1^-/-^ are CRISPR knockout cells were polyclonal after puromycin screening. (C) Expression of Drp1 in Vector, Vector + siDrp1, PINK1^-/-^ and PINK1^-/-^ + siDrp1 analyzed by western blots (n=3 cell protein lysates).
